# GsAPK, an ABA-Activated and Calcium-Independent SnRK2-Type Kinase from *G. soja*, Mediates the Regulation of Plant Tolerance to Salinity and ABA Stress

**DOI:** 10.1371/journal.pone.0033838

**Published:** 2012-03-16

**Authors:** Liang Yang, Wei Ji, Peng Gao, Yong Li, Hua Cai, Xi Bai, Qin Chen, Yanming Zhu

**Affiliations:** 1 Plant Bioengineering Laboratory, Northeast Agricultural University, Harbin, Heilongjiang, China; 2 Fujian Province Key Laboratory of Plant Virology, Institute of Plant Virology, Fujian Agricultural and Forestry University, Fuzhou, Fujian, China; 3 Agriculture and Agri-Food Canada, Lethbridge Research Centre, Lethbridge, Alberta, Canada; Massachusetts Eye & Ear Infirmary, Harvard Medical School, United States of America

## Abstract

Plant Snf1 (sucrose non-fermenting-1) related protein kinase (SnRK), a subfamily of serine/threonine kinases, has been implicated as a crucial upstream regulator of ABA and osmotic signaling as in many other signaling cascades. In this paper, we have isolated a novel plant specific ABA activated calcium independent protein kinase (*GsAPK*) from a highly salt tolerant plant, *Glycine soja* (50109), which is a member of the SnRK2 family. Subcellular localization studies using GFP fusion protein indicated that *GsAPK* is localized in the plasma membrane. We found that autophosphorylation and Myelin Basis Protein phosphorylation activity of GsAPK is only activated by ABA and the kinase activity also was observed when calcium was replaced by EGTA, suggesting its independence of calcium in enzyme activity. We also found that cold, salinity, drought, and ABA stress alter *GsAPK* gene transcripts and heterogonous overexpression of *GsAPK* in *Arabidopsis* alters plant tolerance to high salinity and ABA stress. In summary, we demonstrated that *GsAPK* is a *Glycine soja* ABA activated calcium independent SnRK-type kinase presumably involved in ABA mediated stress signal transduction.

## Introduction

Plants are immobile and continuously exposed to adverse environmental stresses, such as drought, high salinity, and cold, which often imposes a water deficit in plant cells, i.e. osmotic stress. Therefore, plants have evolved complex regulatory mechanisms that act at the level of transcription, post-transcription and/or post-translation in order to reprogram gene expression, protein enzymatic activity leading to adjustment of the cellular milieu and plant tolerance [1]. Some of these stress adaptation responses are mediated by the phytohormone ABA (Abscisic Acid) through complex signal transduction cascades [2].

Protein kinases have been implicated as crucial upstream regulators of ABA and osmotic signaling as in many other signaling cascades. A large number of studies have indicated that water deficit could cause increases in cytosolic Ca^2+^ concentration [3], [4], [5] and calcium-dependent protein kinases (CDPK) were found to be induced and activated by ABA and other stresses in different plant species [6], [7]. Another group of Ca^2+^-regulated protein kinases of key importance in stress signaling are the calcium/calmodulin-dependent protein kinases (CaMKs) that do not directly bind Ca^2+^ by themselves, but instead interact with a specific Ca^2+^ sensor, such as calmodulin (CaM) or calcineurin B-like protein (CBL) [8], [9], [10], [11], [12], [13], [14]. Numerous studies have shown that MAPK cascades are involved in ABA signaling. ABA treatment can activate several MAPK isoforms with molecular masses of ∼40 kD from different plants, such as p45MAPK (*Pisum sativum*) [15], p38MAPK (*Funaria hygrometrica*) [16], AtMPK3, p46MAPK, AtMPK6 and AtMPK4 (*Arabidopsis thaliana*) [17], [18], [19], OsMAPK5 (*Oryza sativa*) [20], and p46MAPK (*Zea mays*) [21], [22].

Plant Snf1 (sucrose non-fermenting-1) related protein kinasea (SnRK) represent a subfamily of serine/threonine kinases that are highly conserved throughout evolution. According to sequence similarity, domain structure and cellular function, the SnRK could be classified as 3 subgroups: SnRK1, SnRK2, and SnRK3. The *SnRK2* and *SnRK3* genes are unique to plants and have 42∼45% amino acid sequence identity with SnRK1 in the kinase catalytic domain [23]. To date, reports indicate that SnRK2 and SnRK3 are implicated to function in ABA and/or abiotic stress signaling. There are 10 *SnRK2* genes and 25 *SnRK3* genes encoded by the *Arabidopsis* genome [24], [25]. *SRK2C*, an *Arabidopsis* SnRK2, has been shown to improve drought tolerance by controlling stress-responsive gene expression [26]. A guard cell specific Ca^2+^-independent and ABA-activated protein kinase, AAPK from *Vicia faba* and its *Arabidopsis* ortholog OST1/SRK2E regulate ABA-induced stomatal closure during drought stress [27], [28], [29], [30]. In rice, 10 members of *SnRK2* gene family were identified and all of them are activated by hyperosmotic stress. Three of these are also activated by ABA. Surprisingly, there were no members that were only activated by ABA [31]. PKABA1 (ABA-responsive protein kinase 1) from wheat also belongs to the SnRK2 family, which is involved in mediating ABA-induced changes in gene expression [32]. Unlike SnRK1 and SnRK2, SnRK3 is calcium-dependent for its interactions with a calcium-binding protein [33]. The *Arabidopsis* SnRK3 family includes SOS2 (salt overly sensitive 2), which functions in ion homeostasis and is involved in conferring salt tolerance [34], [35]. There is biochemical evidence that PKS3, PKS18 or CIPK3, members of the SnRK3 family, modulate ABA sensitivity in seed germination, stomatal closure and seedling growth [9], [33], [36]. Moreover, PKS3 and SOS2 were found to interact with ABA insensitive 2 (ABI2) phosphatase with specificity [33], [37].

In this paper, we use a highly salt tolerant plant *Glycine soja* (50109, from Jilin Academy of Agricultural Sciences, Changchun, China) to isolate salt-tolerance-related genes and for elucidating the stress-signaling network. An up-regulated expressed sequence tag (EST) was identified from previous gene expression data in *Glycine soja* (50109) and the full length sequence was obtained by in silico cloning. We describe a Ca^2+^-independent, ABA-activated protein kinase involved in Ca^2+^-independent ABA signaling pathways. The subcellular localization and expression patterns of *GsAPK* under cold, salt, ABA, and PEG treatments are also characterized. Furthermore, we found that heterogonous overexpression of *GsAPK* in *Arabidopsis* alters plant tolerance to salt and ABA stress.

## Results

### Isolation and sequence analysis of gene *GsAPK*

The overall EST expression patterns of *Glycine soja* under drought, salinity and cold stress were inferred using gene expression profiles of leaves previously established in our laboratory (unpublished data). Sixty-five differentially expressed ESTs annotated as putative kinase were selected and these ESTs are up-regulated under more than one stress treatment. Some novel kinase genes were acquired by in silico cloning wildsoybean ESTs and soybean sequences (Blastall, E value = 1e-20; phrap, minmatch = 14 and minscore = 30). From these, one salinity-up-regulated gene, belonging to the the SnRK2 family was studied. The full-length cDNA was successfully cloned by RT-PCR and we designated it as *GsAPK* (GenBank™ Accession NO. GU062183), which is 1033 bp in length and contains a complete ORF of 1020 bp encoding 340 amino acids with an estimated molecular weight of 38.48 kDa and a theoretical pI of 6.84.

**Figure 1:**
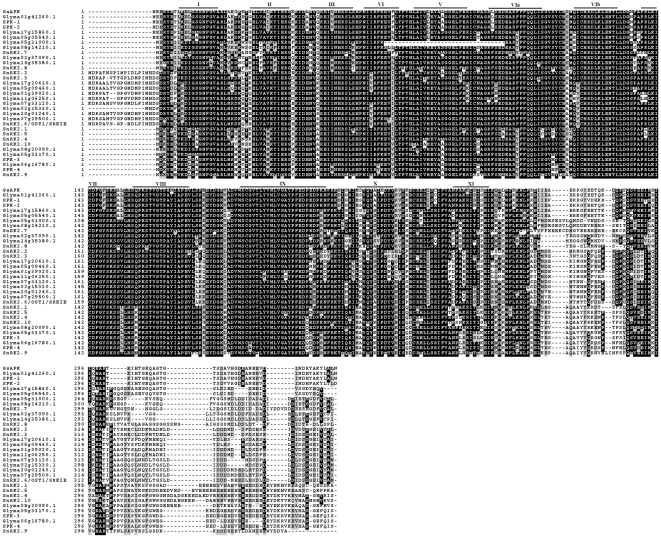
Amino acid sequence alignment of GsAPK and all SnRK2 family members of soybean and *Arabidopsis*. Multiple sequence alignments were conducted using ClustalW (version 1.8.1) and presented using BOXSHADE (version 3.21; http://www.ch.embnet.org/software/BOX_form.html). Identical amino acid residues are boxed, and similar residues are shaded in gray. Dashes indicate gaps in the sequences to allow maximum alignment. The subdomains of catalytic domain are marked by a solid line. *Arabidopsis* Genome Initiative identification numbers of the *Arabidopsis* SnRK2 members are as follows: SnRK2.1 (At5g08590), SnRK2.2 (At3g50500), SnRK2.3 (At5g66880), SnRK2.4 (At1g10940), SnRK2.5 (At5g63650), SnRK2.6/OST1/SRK2E (At4g33950), SnRK2.7 (At4g40010), SnRK2.8 (At1g78290), SnRK2.9 (At2g23030), and SnRK2.10 (At1g60940). OST1/SRK2E is synonymous to SnRK2.6. Nomenclature of the soybean SnRK2 members followed that of the Phytozome v5.0: *Glycine max* database (http://www.phytozome.net/soybean) and the GenBank accession numbers of the sequences from soybean are as follows: SPK-1(AAA33979), SPK-2(AAA34017), SPK-3(AAB68961) and SPK-4(AAB68962).

As shown in Figure 1, the GsAPK protein contains all conserved subdomains of the catalytic domains (KD, amino acids 8 to 255), required for kinase activity [38], [39]. Similar to other Ser/Thr kinases, GsAPK subdomain VIII contains a G-T/S-XX-Y/F-X-APE motif [40], identifying that it as a potential Ser/Thr kinase rather than a Tyr kinase [41]. The KD of GsAPK includes the protein kinase ATP binding region I and a Ser/Thr kinase active site signature (ICHRDLKLENTLL, residues 120 to 132). A highly conserved Thr residue between the DFG (domain VII) and APE (domain VIII) conserved motifs that define the borders of the activation domain segment, was identified. Phosphorylation of this motif has been reported to activate many other protein kinases [42]. In addition, GsAPK is an RD-type protein kinase because it has the Asn residue in subdomain VIb (D-124) and an Arg immediately preceding this Asn residue [43]. Other than the conserved kinase domain, GsAPK also contains a consensus plant N-myristoylation motif [MGXXXS/T(K/R)] [44], [45], suggesting membrane localization because myristoylation is involved in directing and anchoring proteins to membranes [46].

**Figure 2:**
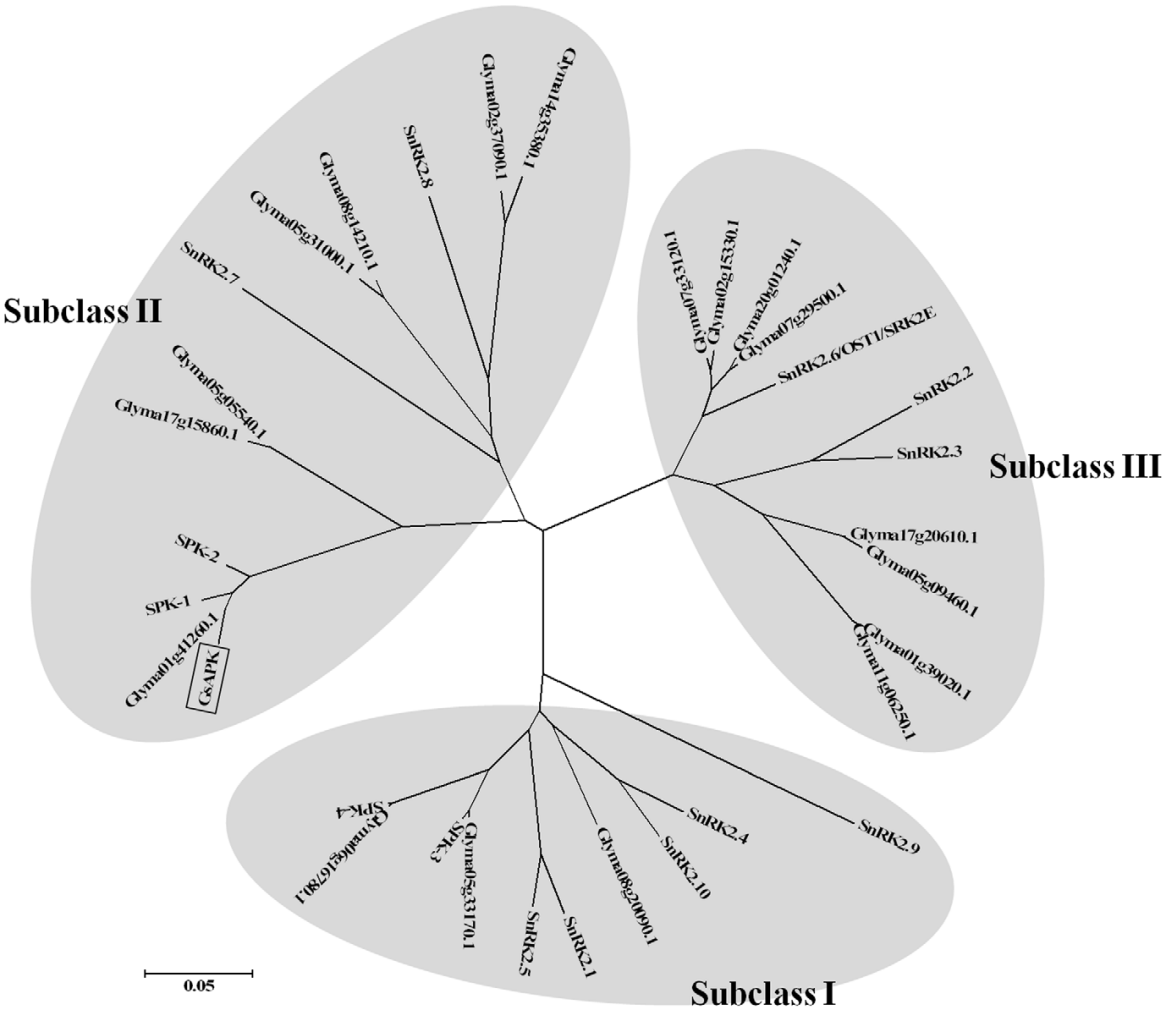
The phylogenetic relationship of GsAPK and all SnRK2 family members of soybean and *Arabidopsis*. An unrooted neighbor-joining tree was built using MEGA 4.0. Sequences and phylogenic data are as described in Figure 1.

A phylogenetic tree was constructed using MEGA 4.0 [47] based on the amino acid sequences of GsAPK and all the members of the soybean and *Arabidopsis* SnRK2 family. By searching genome sequence databases (http://www.phytozome.net/soybean), 22 genes were identified that encode SnRK2 family protein kinases in the soybean genome. Ten SnKR2 family proteins has been reported to be encoded in the *Arabidopsis* genome [28].The amino acid sequences of the SnRK2 protein can be divided into two parts, the N-terminal highly conserved kinase domain and the divergent C-terminal domain containing regions rich in acidic amino acids [48]. As shown in Figure 2, the tree suggests that these protein kinases can be divided into three subclasses, denoted here as subclasses I, II, and III. Because each subclass contains *Arabidopsis* members, the distinction of the subclasses is believed to have been established before the divergence of dicot and monocot phyla. *Arabidopsis* SnRK2.6 (OST1/SRK2E), which has been reported to be activated by ABA and involved in the ABA regulation of stomatal closing, are included in subclass III. Halford and Hardie [48] have divided the family into SnRK2a (corresponding to subclass I) and SnRK2b (corresponding to subclass II) in a comparison that does not include the subclass III members. More structural similarities were found between subclasses II and III than other pairs: (1) the number of amino acids is fewer compared with subclass I; and (2) the acidic patches are rich in Asp, whereas those of subclass I are abundant in Glu (Figure 1). The last difference was previously pointed out as a difference between SnRK2a and SnRK2b [48], indicating that subclass III can be included in SnRK2b. GsAPK is highly homologous to Glyma01g41260.1 that is more homologous to SPK-1 than to SPK-2 (Figure 2).

### Expression of GsAPK gene is regulated by multiple abiotic stresses

**Figure 3:**
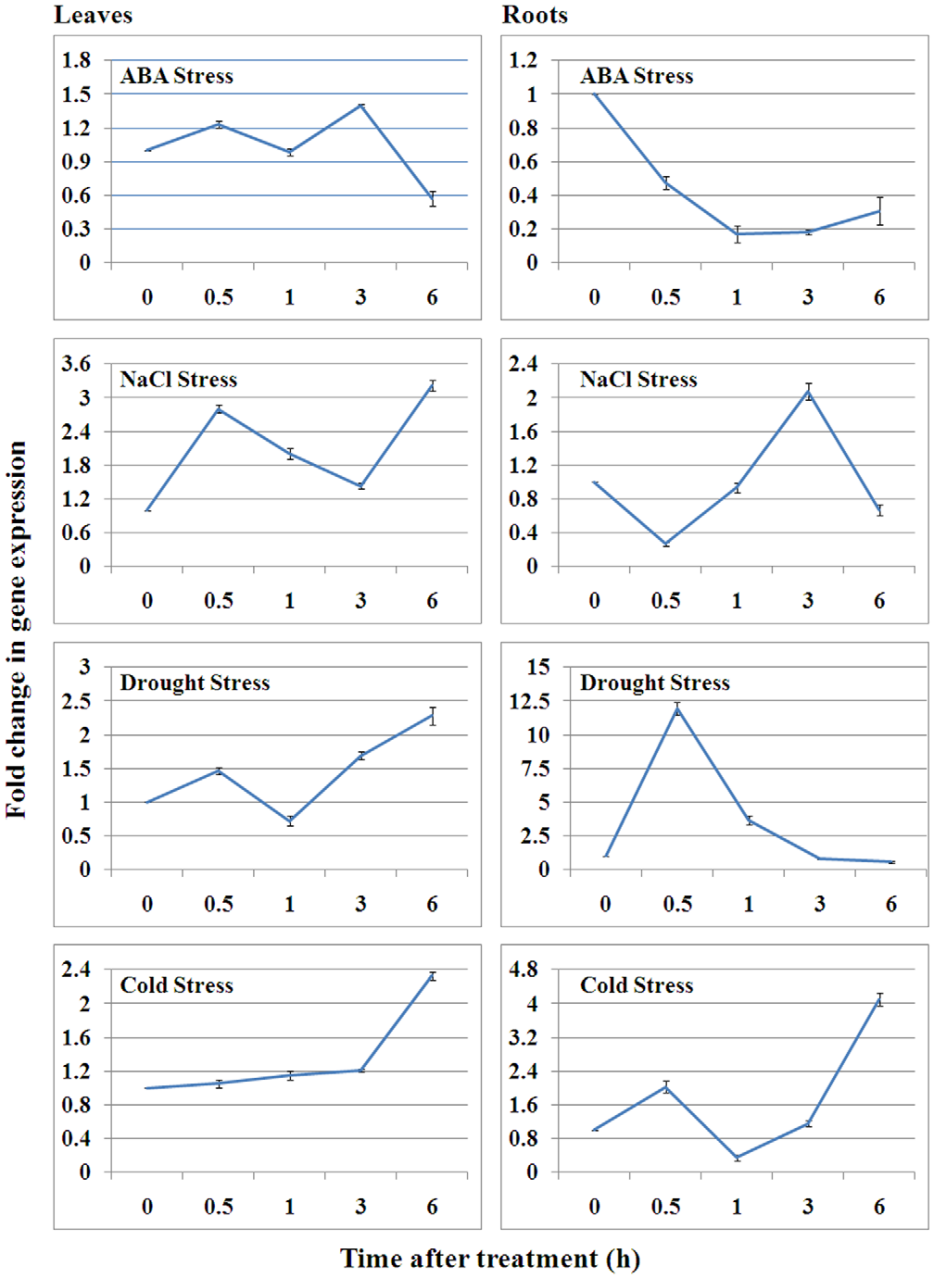
Expression pattern of *GsAPK* gene in *Glycine soja* after exposure to various abiotic stresses. Total RNA was extracted from leaves or roots of one-month-old *Glycine soja* exposed to various stress treatments. Relative transcript levels were determined by real-time PCR according to the 2^−ΔΔC^_T_ method [57] using *actin* gene as an internal control. Gene expression was normalized to the unstressed expression level, which was assigned a value of 1. Data represent the average of three independent biological experiments ± S.E. ABA treatment, 100 µM ABA; Salt treatment, 200 mM NaCl; Drought treatment, 30% (W/V) PEG 6000; Cold treatment, 4°C.

Real-time PCR analyses were performed to illustrate the responses of *GsAPK* gene expression patterns after different abiotic stresses (Figure 3). In leaves, the transcripts of *GsAPK* were constant from 0.5 h to 3 h and then increased at 6 h after cold treatment. Conversely, *GsAPK* was down-regulated at 6 h after application of exogenous ABA. Increased transcripts of *GsAPK* were detected after both NaCl and PEG treatments at 0.5 h. Under NaCl treatment, the transcripts began to decline gradually from 1 h before reaching a maximum level at 6 h. While, for PEG response, reduced transcript levels were observed at 1 h and then returned back to the level at 0.5 h. In roots, the transcripts followed a more complex pattern as illustrated in Figure 3. Under cold treatment, increased transcripts of *GsAPK* was detected at 0.5 h, followed by reaching the lowest level at 1 h and then increased gradually. *GsAPK* was down-regulated and reached trough level at 1 h after treatment of ABA. For NaCl response, the *GsAPK* transcript obviously declined at 0.5 h and returned back to normal afterwards and then increased at 3 h. 30% (w/v) PEG 6000 used for imitating drought stress, the transcript was strongly up-regulated and reached the highest level at 0.5 h.

### GsAPK protein is localized to the plasma membrane of plant cells

**Figure 4:**
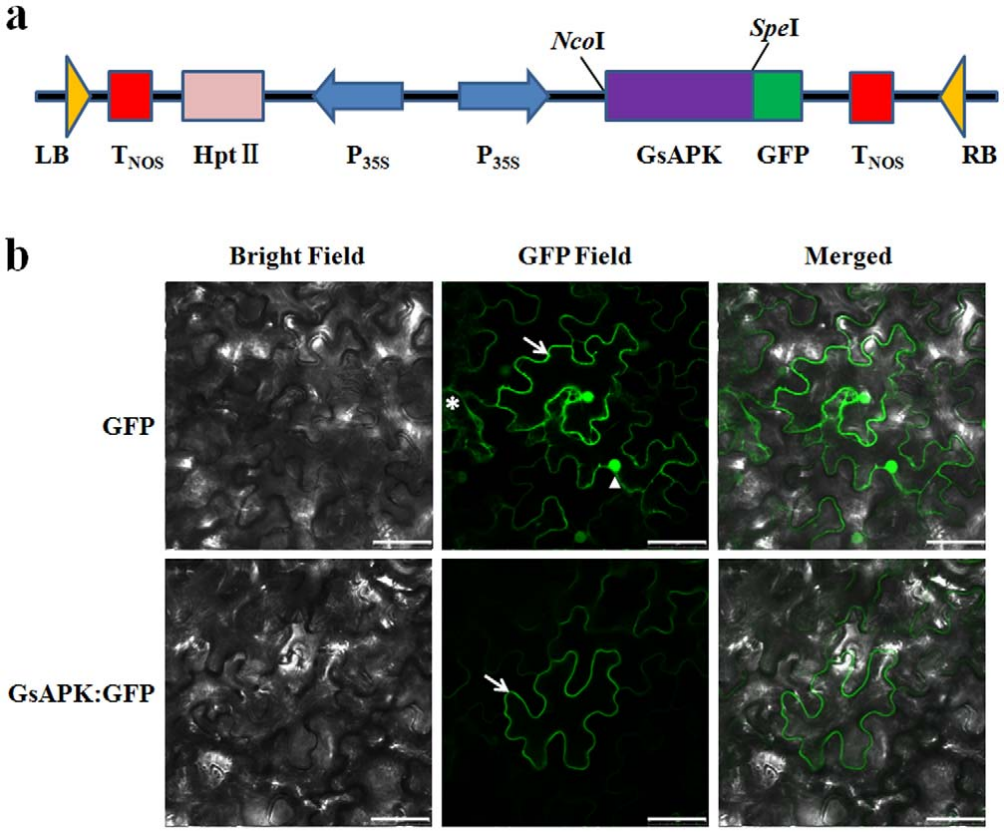
GsAPK protein targets to the plasma membrane of *N.benthamiana* plant cells. **a.** Schematic representation of construct used for subcellular localization of the GsAPK protein. **b.** Subcellular localization assay of the GsAPK:GFP fusion protein in *N. benthamiana* epidermal cells. Micrographs showing cells expressing GFP (control, upper lane) or GsAPK:GFP (bottom lane) fusion protein, which were examined under bright-field illumination (left), and under fluorescent-field illumination (middle) to examine GFP fluorescence, and by confocal microscopy (right) for an overlay of bright and fluorescent illumination. Scale bar, 50 µm. Arrow, plasma membrane; asterisk, cytosol; triangle, nucleus.

To better understand the mechanisms of GsAPK function, the subcellular localization of the GsAPK protein was investigated using the green fluorescent protein (GFP) dependent method. The *GsAPK* coding sequence was fused in-frame to the 5′end of the mGFP5 (Figure 4a) and the recombinant fluorescent protein was transiently expressed in tobacco leaf cells via agrobacterium infiltration. Because N-myristoylated proteins are often translocated to the membrane [46], we expected the GsAPK protein to be localized to the membrane. The GsAPK:GFP fusion protein was observed solely at the plasma membrane, otherwise cells expressing GFP alone showed the GFP signal around the nucleus and in the cytosol (Figure 4b).

### GsAPK is a ABA-activated protein kinase in a Ca^2+^-independent manner

**Figure 5:**
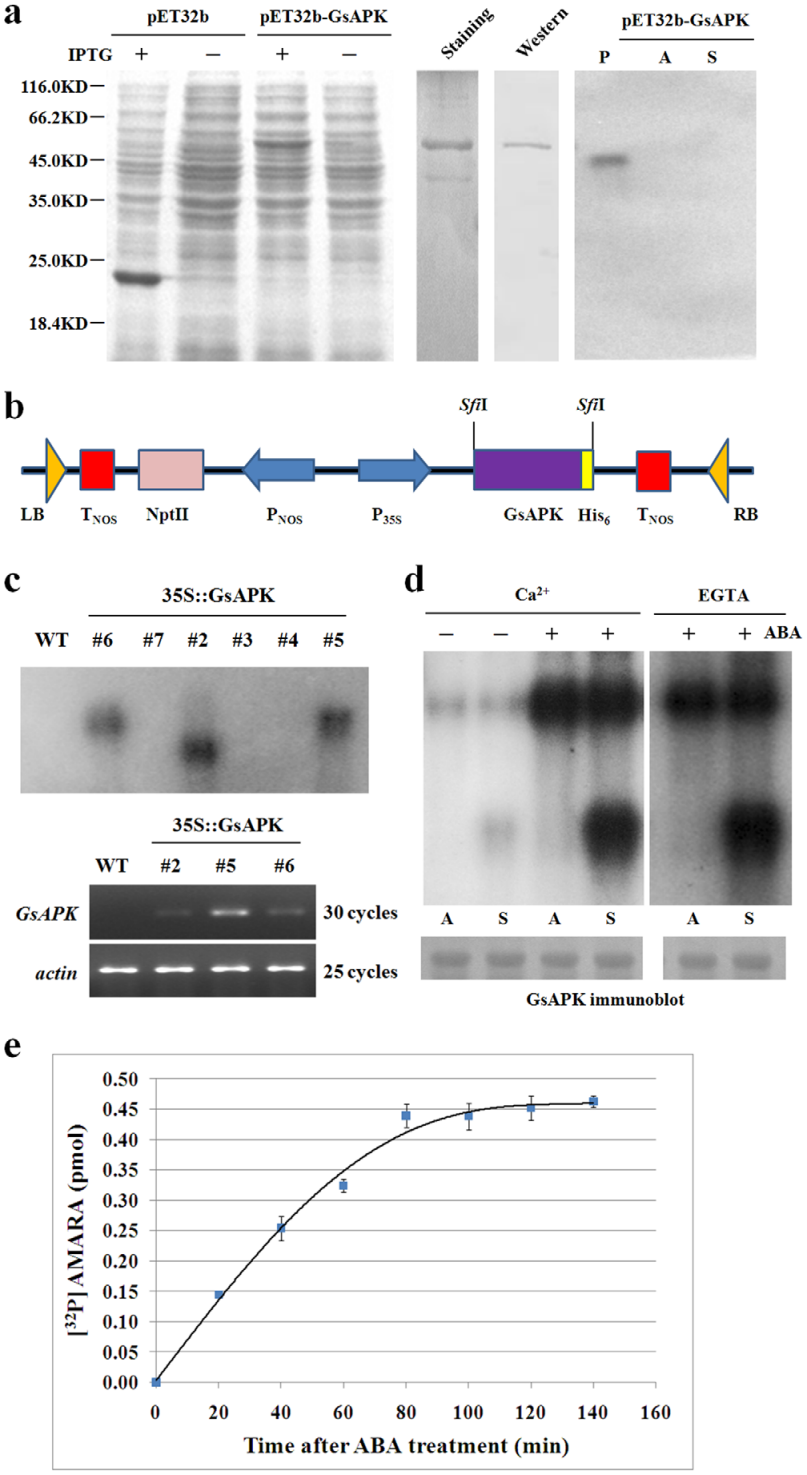
Enzymatic properties of protein GsAPK. MBP was used as the general substrate. P, positive control (autophosphorylation). A, autophosphorylation. S, substrate phosphorylation. **a.** Phosphorylation activity assay of GsAPK produced in *E.coli*. The total proteins from *E. coli* strains containing pET32b-GsAPK or pET32b empty vector were separated by SDS-PAGE. The purified recombinant GsAPK protein confirmed by western blot analysis using the anti-HIS_6_ antibody was subjected to phosphorylation assays, while displaying no detectable kinase activity in vitro assay. **b.** Schematic representation of a construct was used for *Agrobacterium tumefaciens*-mediated transformation which could overexpress the HIS_6_ tag-fused GsAPK protein. **c.** Identification of the *GsAPK* genes in the transgenic lines. DNA gel blot verifying the presence of *GsAPK* gene in the three independent T_4_ generation transgenic lines (upper lane). Transgenic *Arabidopsis* genomic DNA was digested with *EcoR*I and probed with the coding region of *GsAPK* gene. Wild type plant was performed as negative control. Expression of *GsAPK* genes in wild-type (WT) and three T_4_ generation transgenic lines (bottom lane). Total RNA extracted from 2-week-old seedlings were analyzed by semi-quantitative RT-PCR with a*ctin* gene as an internal standard. **d.** Effect of ABA on regulating the auto and substrate phosphorylation of protein GsAPK. The protein was extracted from 2-week-old transgenic *Arabidopsis* roots treated for 3 h with 10 µM ABA (+) or without treatment (−). EGTA was added in the kinase buffer replacing CaCl_2_ to verify whether the kinase activity is in a Ca^2+^ dependent manner. **e.** Time course substrate phosphorylation activity of GsAPK after ABA treatment. AMARA peptides were phosphorylated by GsAPK for the indicated times and [^32^P] AMARA were counted in a liquid scintillation counter. Data represent the means (±S.E.) from three independent experiments.

To examine whether GsAPK is a functional protein kinase, we performed a phosphorylation assay using HIS_6_-fused GsAPK protein produced in *E.coli*. The purified recombinant protein migrated at the expected molecular mass upon gel electrophoresis while displaying no detectable kinase activity in vitro (Figure 5a). Western blot analysis using the anti-HIS_6_ antibody was performed to confirm the identity of GsAPK (Figure 5a). Similar result in vitro was also observed in *V. faba* AAPK and *Arabidopsis* SRK2E and they are activated by ABA [27], [28], [29], suggesting that protein GsAPK might be active only upon stimulation of the ABA signaling cascade in planta. Thus, we tested whether the kinase activity of GsAPK was activated by ABA. To do this, transgenic *Arabidopsis* plants were generated that could overexpress GsAPK as a fusion protein with polyhistidine affinity (His) tags (Figure 5b) and the recombinant protein was extracted from 2-week-old transgenic *Arabidopsis* roots treated for 3 h with 10 µM ABA and was purified to detect kinase activity. As shown in Figure 5d, in both autophosphorylation and substrate phosphorylation, activity was markedly enhanced in response to ABA, whereas essentially slight kinase activities were detected when the plants were subjected to control treatment. The kinase activity was also observed when calcium was replaced by EGTA in the assay. This indicates that GsAPK is an ABA-activated Ca^2+^-independent protein kinase. Time-course analysis revealed that AMARA substrate phosphorylation activity of GsAPK was already detectable after 20 min and reached near maximal levels after 120 min of ABA treatment (Figure 5e).

### Overexpression of GsAPK in *Arabidopsis* alters plant tolerance to salinity and ABA

To further characterize the function of *GsAPK*, we generated *Arabidopsis* transgenic plants which overexpressed *GsAPK* gene under the control of the strong constitutive CaMV35S promoter (Figure 5b). Three independent T4 generation lines transgenic lines were identified by DNA gel blot and semi-quantitative RT-PCR (Figure 5c). These results showed that each of these three lines expressed *GsAPK* in plants.

**Figure 6:**
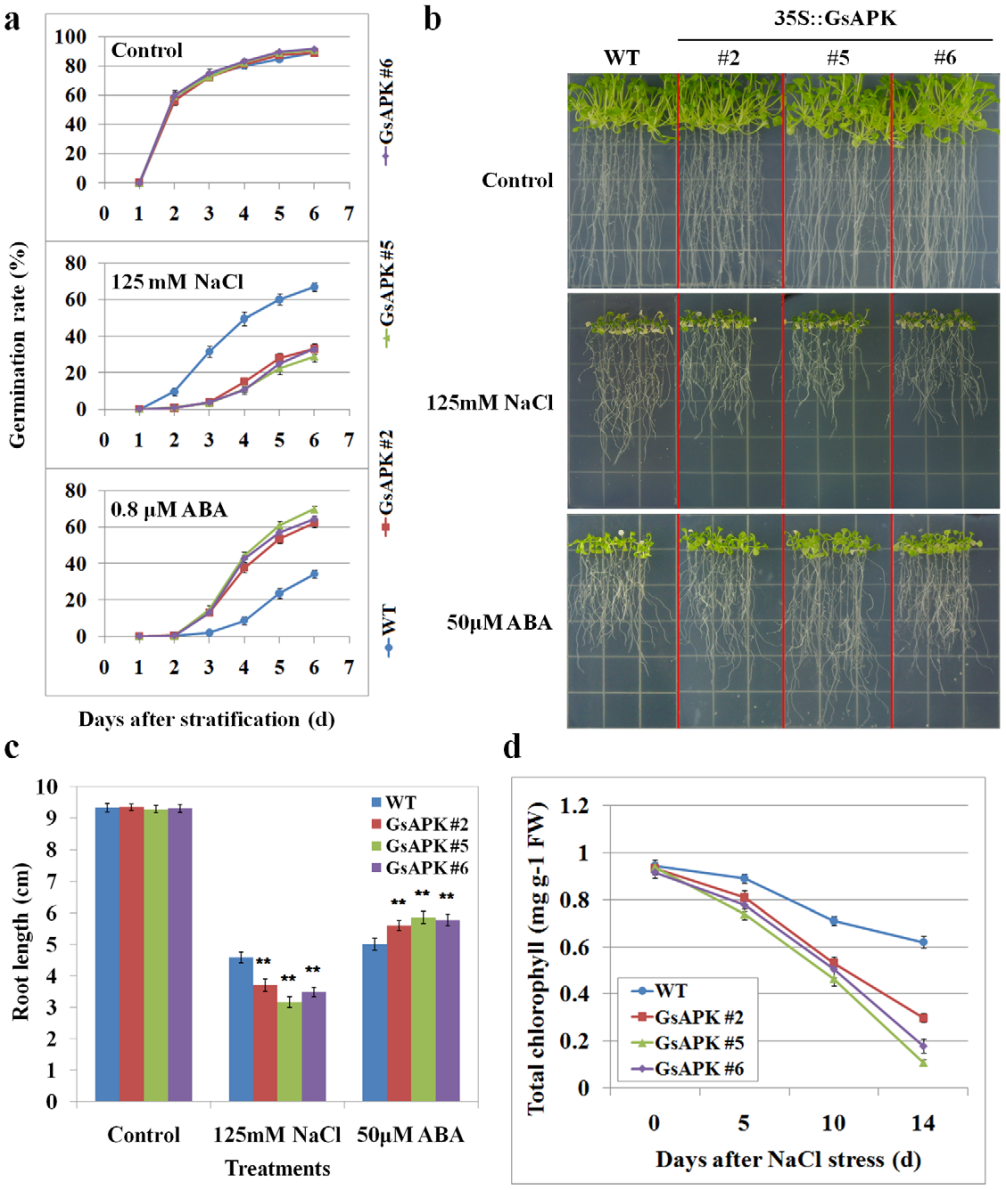
Phenotypic analysis of transgenic *Arabidopsis* plants under salinity stress and ABA treatments. **a.** Response of seed germination to ABA and NaCl in transgenic and wild-type *Arabidopsis* plants. Seeds from three transgenic and wild-type *Arabidopsis* were stratified on 0.5× MS agar plates in the absence or presence of 100 mM NaCl or 0.8 µM ABA, respectively. Data are means (±S.E.) of five replicates (each with 100 seeds for each line). **b.** Phenotypes of wild-type and three transgenic seedlings grown in control or stress condition. 7-day-old WT and transgenic seedlings grown on 0.5× MS were transferred to new solid agar plates supplemented with 125 mM NaCl or 50 µM ABA, respectively. Photographs were taken after 12 days growth. **c.** Measurements of root lengths of plants shown in b. **d.** Time course total chlorophyll content of wild-type and three transgenic seedlings under NaCl stress. Three-week-old soil-grown plants were irrigated with 200 mM NaCl for 2 weeks and the total chlorophyll content among the wild-type and three transgenic plants were measured. All values in c and d are means (±S.E.) from three independent experiments (30 seedlings per experiment). **: *P*<0.01 by Student's *t* test.

Under standard culture conditions, we did not observe a noticeable difference on the germination of three T_4_ generation transgenic lines (#2, #5 and #6) over-expressing GsAPK compared with wild types (WTs). In the presence of 100 mM NaCl, the germination of seeds from the three GsAPK over-expressor lines was less successful than in the WT seeds, whereas the higher germination of the three GsAPK over-expressor lines compared to the WT seeds was observed by exposure to 0.8 µM ABA (Figure 6a).

Similar results were observed at the seedling stage. Root growth assay was conducted to measure the physiological change between the transgenic and wild-type seedlings. As shown in Figure 6b, the growth of primary roots was significantly less inhibited in the WT plants compared to that of GsAPK over-expressor lines under NaCl stress. Conversely, in ABA treatment, root length of transgenic *Arabidopsis* plants is longer than WT plants. Statistical analysis (Figure 6c) confirmed that over-expressing *GsAPK* can significantly alter plant tolerance to salinity and ABA stress.

When 3-week-old soil-grown plants were irrigated with 200 mM NaCl for 2 weeks, the total chlorophyll (Chl.) content among the wild-type and three transgenic plants were monitored. Without NaCl treatment, there was almost no difference in growth and total chlorophyll content among the wild-type and three transgenic plants. However, in the presence of NaCl stress, the total chlorophyll content decreased upon salt stress in both the transgenic and wild-type plants and the extent of this decline in transgenic plants was more than that in wild-type plants (Figure 6d).

## Discussion

Plant protein kinases play crucial roles in signal perception, amplification and transduction under abiotic and biotic stress. To date, numerous SnRK members have been identified from diverse plant species and demonstrated to be activated by hyperosmotic stress and/or ABA signal in vivo. Here, we have isolated full-length *GsAPK* as a salt-inducible gene by differential screening which encodes a ser/thr protein kinase belonging to the SnRK family (Figure 1). We also characterized detailed biochemical properties of the GsAPK protein by in vitro kinase assay. We demonstrated that the bacterial extracts from *E. coli* did not have the active SnRK2 enzyme (Figure 5a), possibly due to lack of specific upstream kinase required for enzyme activation. In contrast, GsAPK showed strong enzyme activity to phosphorylate itself and exogenous substrates as well only upon stimulation of the ABA signaling cascade in transgenic *Arabidopsi*s plants (Figure 5d, e) and similar results were also reported in the previous trials [27], [28], [49], [50].

Chae et al. [51] reported that the kinase activity of GST-OSRK1 was completely abolished when rice leaf extract was added to the assay buffer, suggesting that inhibitors of OSRK1 activity may be present in a plant cell. It was not identified whether in vivo OSRK1 activity is under phosphorylation-mediated negative regulation by protein phosphatases or inhibiting kinases. Park et al. [52] revealed new insights into ABA signalling mechanisms. They demonstrated that the default state of the SnRK2 kinases is an autophosphorylated, active state, and are kept inactive by the PP2Cs through physical interaction and dephosphorylation. However, in the presence of ABA, the PYR/PYL (pyrabactin resistance 1/PYR1-like) receptor proteins, which were the recently identified ABA receptors, can disrupt the interaction between the SnRK2s and PP2Cs, thus preventing the PP2C-mediated dephosphorylation of the SnRK2s, leading to the activation of the SnRK2 kinases. In our study, GsAPK, a member of SnRK2s, shows strong kinase activity activated by ABA and may be involved in the same mechanism of the ABA-activated regulation as *Arabidopsis* SnRK2s. Moreover, the kinase activity also was observed when calcium was replaced by EGTA in the assay. This indicates that GsAPK is an ABA-activated Ca^2+^-independent protein kinase. At present, phosphorylation sites and physiological functions for the autophosphorylation of GsAPK are unknown. It may be involved in the activity regulation or autoactivation of GsAPK and may result in complicated phosphorylation. Further molecular and biochemical studies are needed in order to fully elucidate whether our recombinant GsAPK protein is also in the functionally heterologous mutiphosphorylation state and reveal the action mode of ABA modulated protein GsAPK activity.

Protein kinases are localized in diverse cellular compartments for their different functions. Although the plant SnRK family has been implicated as a crucial component of hyperosmotic and ABA signaling pathways, the subcellular localizations of most SnRK family members are unknown. GsAPK contains N-terminal myrisotylation motif that is a recognizable targeting sequence. Previous reports showed that N-myristoylation is considered crucial in plant signal transduction in response to environmental stress and disease resistance [53], [54]. Based on the confocal laser scanning microscope observation of tobacco epidermal cell transiently expressed GFP-fused GsAPK (Figure 4), we suggest that GsAPK is a plasma membrane localized kinase. Our data differ from the previous reports: fava bean AAPK is present in the guard cell nucleus and modulates a nuclear RNA binding protein by ABA-induced phosphorylation [27]. TtPK1. A wheat SnRK2 was targeted to the nucleus and cytosol [55]. More recently, Chae et al. [51] reported that OSRK1 is a nuclear localized kinase and can be distributed to the plasma membrane and cytosol as well. The reason for this diversity may depend on the specific signals or interacting proteins in a highly regulated manner and confer various functions to SnRK-type kinases.

*GsAPK* is regulated by cold, drought, salt stresses, and the hormonal molecule ABA (Figure 3). There are at least two independent signal transduction pathways in plants under abiotic stresses: ABA-independent and ABA-dependent signal transduction cascades. Expression pattern analysis and kinase activity assay indicated that *GsAPK* is possibly involved in an ABA-dependent signal transduction pathway.

We also demonstrated that over-expression of *GsAPK* in transgenic plants allowed better seed germination and root development than that in wild type under ABA stress (Figure 6) while the converse situation occurs in salinity stress. It is likely that *GsAPK* responds to environmental or hormonal signals in two ways: by altering the mRNA expression level, and by stimulating its kinase activity in response to the changes of ABA concentrations triggered by environmental stimulus. Based on the previous reports [51]–[52], [56]–[60], the possible molecular mechanism of GsAPK function is that, under ABA treatment, the inhibition of the negatively acting PP2Cs leads to the successful activation of GsAPK, which phosphorylate the basic leucine zipper (bZIP) transcription factors called ABFs/AREBs. The ABFs bind to ABA-responsive promoter elements (ABRE) to induce the expression of ABA-responsive genes and then confer the resistance of transgenic *Arabidopsis* in seed germination and root elongation. While, in the presence of NaCl stress, it may trigger not only the ABA response pathway, but also some unknown signalings. The phenotypic changes of overexpressors are the result of these two signal transductions and further molecular and biochemical investigations are needed to fully reveal the action mode of ABA modulated protein GsAPK activity and characterize the complex cross-talk of plant stress responses.

## Materials and Methods

### Plant growth and stress treatments

Wild soybean (*Glycine soja*) cultivar (50109, from Jilin Academy of Agricultural Sciences, Changchun, China) was grown in vermiculite in a greenhouse with 15 h light/9 h dark. The roots of one-month-old seedlings were submerged in a 0.5× Murashige and Skoog (MS) solution (pH5.8) containing either 200 mM NaCl or 30% (w/v) PEG 6000 or 100 µM ABA. Low temperature treatments were applied by transferring plants to 4°C environment and equal numbers of leaves and roots tissues were harvested at 0.5 h, 1 h, 3 h and 6 h. Control was performed without treatment, mixed from different time points and named 0 h. All samples were immediately frozen in liquid nitrogen, and stored at −80°C for RNA extraction.

Wild-type (WT) *Arabidopsis thaliana* (Columbia ecotype, from Northeast Agricultural University, Harbin, China) plants used for transformation were grown in the greenhouse under controlled environmental conditions (21 to 23°C, 100 µmol photons m^−2^ s^−1^, 60% relative humidity, 16 h light/8 h dark).

### Isolation of *Glycine soja* osmotic stress-related-kinase gene

*G. soja* ESTs were downloaded from dbEST database (http://www.ncbi.nlm.nih.gov/dbEST/index.html), and the expression patterns of ESTs under drought, salinity and cold stress were inferred from *Glycine soja* leaf gene expression profiles previously established in our laboratory (unpublished data). The up-regulated ESTs annotated as putative kinases under more than one stress treatment were selected, and then, some novel kinase genes were acquired by in silico cloning wildsoybean ESTs and soybean sequences (Blastall, E value = 1e-20; phrap, minmatch = 14 and minscore = 30). Among these, one salinity-up-regulated gene, GsAPK, was chosen for further study. Two primers designed according to the full-length cDNA sequence were described as follows: (5′-GGTCTGTAATTTTATTGCTTTTGGTG-3′) and (5′-AAGAATGCACACTAGCTTTGGTAAAC-3′). The PCR products were cloned into pGEM-T cloning vector (Promega, Madison, WI), and then subjected for sequencing.

### RNA Isolation, cDNA Preparation, Primer Design, and Quantitative Real-Time PCR

Total RNA was extracted from control and stress-treated tissues (described above) using RNeasy Plant Mini Kit (Qiagen, Valencia, CA, USA). Two micrograms of DNase treated total RNA was reverse transcribed to cDNA using the superscript III Kit (Invitrogen, Carlsbad, CA, USA) with oligo d(T)_18_ reverse primer following the manufacturer's instructions.

The primer pairs for amplifying *GsAPK* (5′-AGCATCTCGGAGATAAAACAGCAC-3′; 5′-TGATCCGCATGATTTCTTCCAC-3′) and *actin* (5′-GAAGATGGCAGACGCTGAGGAT-3′; 5′-ACGACCTACAATGCTGGGTAACAC-3′) were designed using the Primer Express 2.0 software (Applied Biosystems). Real-time PCR was performed according to Kant et al. [61]. The relative quantification values for each target gene were calculated by the 2^−ΔΔC^_T_ method using *actin* as an internal reference gene for comparing data from different PCR runs or cDNA samples [62].

### GsAPK fusion protein expression and purification

In order to detect the kinase activity of protein GsAPK, full-length *GsAPK* was amplified using the following primer pairs: (5′-TTAGGTACCATGGAGGAACGGTACG-3′) and (5′-ATAGTCGACATTAAGGGCTAGGTATTTGG-3′). After digestion with *Kpn*I and *Sal*I, the PCR fragment was cloned into the pET-32b expression vector (Novagen, Madison, WI) to produce the HIS_6_ tag-fused GsAPK. The recombinant GsAPK protein was induced by 1 mM isopropyl 1-thio-β-D-galactopyranoside (IPTG) for 6 h at 20°C and purified over ProBond™ Nickel-Chelating Resin as instructed by the ProBond Purification System manufacturer.

Two-week-old transgenic *Arabidopsis* roots treated for 3 h with 10 µM ABA, then three-gram-plant tissue was collected and ground in 4 volumes/g fresh weight of extraction buffer (50 mM phosphate, pH 8.0, 10 mM Tris, pH 8.0, 500 mM NaCl, 0.1% Tween 20, 0.1% Nonidet P-40, 0.1% β-mercaptoethanol, 1 mM phenylmethylsulfonyl fluoride). His_6_ tagged GsAPK protein was then purified from the extract as described by Lindbo [63].

### Subcellular localization of GsAPK protein

For the analysis of the subcellular localization of GsAPK protein using the fluorescent protein reporter gene, the *GsAPK* gene coding sequence was amplified with primer pair (5′-CCATGGAGATGGAGGAACGG-3′ and 5′-ACTAGTCAGATCTACCATATTAAGGGCTAG-3′) and cloned into the *Nco*I/*Spe*I-digested pCAMBIA1302, which is a N-terminal GFP fusion vector. The intact leaves of 4-week-old *N. benthamiana* plants were infiltrated with *A. tumefaciens* strain EHA105 harboring pCAMBIA1302 and pCAMBIA1302-GsAPK:GFP respectively. By 3 dpi, the localization of fluorescent proteins in the leaves was observed at 488 nm using a confocal laser scanning microscope (SP5; Leica, Germany).

### *In vitro* kinase assays

The kinase assay was performed in 20 µL phosphorylation buffer containing 25 mM HEPES (pH 7.5), 0.5 mM DTT, 10 mM MgCl_2_, 20 µM ATP, 5 µCi of [γ-^32^P] ATP (5000 Ci mmol^−1^) with 1 mM CaCl_2_ or 5 mM EGTA. Myelin Basic Protein (MBP) was added for substrate phosphorylation assay at 30°C for 60 min. The reactions were stopped by adding 5× SDS loading buffer and analyzed by 12.5% SDS-PAGE. The gel was exposed to X-ray film at −80°C and visualization by autoradiography. For the AMARA peptide substrate phosphorylation assay, 450 µM AMARA peptide was added in the reaction mixture. After SDS-PAGE and drying onto Whatman DE81 ion-exchange paper, the [^32^P] incorporated AMARA peptides were cut out and determined by liquid scintillation counter.

### Transformation of *Arabidopsis*

To obtain HIS_6_-fused-GsAPK expressed in *Arabidopsis* plants, the coding region of *GsAPK* was amplified from pET32b-GsAPK (described above) with primers (5′-GGCCATTACGGCCATATGCAC-3′) and (5′-GGCCGAGGCGGCCCCAAGGGG-3′). Restriction-digested PCR product was cloned into a modified version of the binary vector pBHT-5 [64] under the control of separate CaMV35S promoters between the *Sfi*I sites. The construct was introduced into *Agrobacterium tumefaciens* strain LBA4404 and then used for *Arabidopsis thaliana* (Columbia ecotype) transformation through the floral dip method [65]. Transformants were selected on 0.5× MS medium containing 50 µg mL^−1^ kanamycin under controlled environmental conditions (21 to 23°C, 100 µmol photons m^−2^ s^−1^, 60% relative humidity, 16 h light/8 h dark).

### Phenotypic analysis of transgenic *Arabidopsis* plants

For the germination assay, seeds from wild type and T_4_ generation transgenic *Arabidopsis* were surfaced-sterilized, and then sown in 0.5× MS agar medium with 1%(w/v) sucrose, and 0.8% (w/v) agar (supplemented with none or 100 mM NaCl or 0.8 µM ABA, respectively).

For the root length assay, seeds from wild type and T_4_ generation transgenic *Arabidopsis* were germinated on 0.5× MS agar medium for 7 d, followed by transfer to fresh medium (in the absence or presence of 125 mM NaCl or 50 µM ABA, respectively) for vertical growth for 12 days before photography and then root length was measured.

Salt tolerance assay was performed with 3-week old plants grown in soil in a growth chamber under controlled environmental conditions (21 to 23°C, 100 µmol photons m^−2^ s^−1^, 60% relative humidity, 16 h light/8 h dark). Plants were then irrigated with 200 mM NaCl solution every 3 days, and were subsequently monitored for chlorophyll content for the next 2 weeks. The total chlorophyll content was determined spectrophotometrically according to the method described by Arnon [66]. All experiments were repeated at least three times, and the results from one representative experiment are shown. The numerical data were subjected to statistical analyses using EXCEL 2007.
